# The Influence of Cognitive Biases and Financial Factors on Forecast Accuracy of Analysts

**DOI:** 10.3389/fpsyg.2021.773894

**Published:** 2022-01-04

**Authors:** Paula Carolina Ciampaglia Nardi, Evandro Marcos Saidel Ribeiro, José Lino Oliveira Bueno, Ishani Aggarwal

**Affiliations:** ^1^Accounting Department, School of Economics, Business Administration and Accounting, University of São Paulo, USP, Ribeirão Preto, Brazil; ^2^Administration Department, School of Economics, Business Administration and Accounting, University of São Paulo, USP, Ribeirão Preto, Brazil; ^3^Department of Psychology, School of Philosophy, Science and Letters, University of São Paulo, USP, Ribeirão Preto, Brazil; ^4^Brazilian School of Public and Business Administration, FGV, Rio de Janeiro, Brazil

**Keywords:** analysts’ accuracy, analysts’ forecast, cognitive biases, text analysis, random forest

## Abstract

The objective of this study was to jointly analyze the importance of cognitive and financial factors in the accuracy of profit forecasting by analysts. Data from publicly traded Brazilian companies in 2019 were obtained. We used text analysis to assess the cognitive biases from the qualitative reports of analysts. Further, we analyzed the data using statistical regression learning methods and statistical classification learning methods, such as Multiple Linear Regression (MRL), *k*-dependence Bayesian (*k*-DB), and Random Forest (RF). The Bayesian inference and classification methods allow an expansion of the research line, especially in the area of machine learning, which can benefit from the examples of factors addressed in this research. The results indicated that, among cognitive biases, optimism had a negative relationship with forecasting accuracy while anchoring bias had a positive relationship. Commonality, to a lesser extent, also had a positive relationship with the analyst’s accuracy. Among financial factors, the most important aspects in the accuracy of analysts were volatility, indebtedness, and profitability. Age of the company, fair value, American Depositary Receipts (ADRs), performance, and loss were still important but on a smaller scale. The results of the RF models showed a greater explanatory power. This research sheds light on the cognitive as well as financial aspects that influence the analyst’s accuracy, jointly using text analysis and machine learning methods, capable of improving the explanatory power of predictive models, together with the use of training models followed by testing.

## Introduction

The economic development of a country can be influenced by the capital market (Jyothi and Rajyalakshmi, 2019), which is capable of providing better financing conditions for companies, allowing their growth (Rauf-Animasaun et al., 2018). In addition, it is responsible for generating numerous benefits for companies, like trading in loans such as bonds, stocks, and mortgages, channeling surplus funds from investors to those with deficits (Jyothi and Rajyalakshmi, 2019), and a more efficient allocation of resources (Wang et al., 2019; Bae et al., 2021). However, the functioning of the capital market occurs among participants with different interests and with asymmetric access to information. For example, as predicted by the agency theory (Jensen and Meckling, 1976), fundraising companies know more about their economic and financial reality than other companies and are able to influence the information that is made available to investors. In this context of uncertainty, conflicting interests, and information asymmetry, financial analysts, with the capacity to weigh information (Bildstein-Hagberg, 2003), to interpret market indexes (Diakomihalis, 2011), and to assess the disclosed information (Bildstein-Hagberg, 2003), can assist in the optimization of investor decisions and reducing information asymmetry by predicting the results. Specifically, in the capital market, financial analysts are able to assist in investment decisions and work to reduce information asymmetry between investors and companies (Amiram et al., 2017). As investors tend to be influenced by the analysts’ recommendations, their forecasts end up affecting the informational environment and influencing the values of the assets (Kothari, 2001). Hence, an understanding of the degree of accuracy of financial analysts’ forecasts and their predictors is pivotal to improving the valuation models of companies, consequently for a more efficient allocation of resources in the capital market.

In the study of the behavior of financial analysts, decision makers have often been considered as rational decision makers. For example, to explain stock prices through statistical models, important theories were developed and proposed by Sharpe (1964) and Fama (1970), who consider that decision makers are rational and incorporate, in the stock value, the available information. However, the seminal work by Simon (1955) and Kahneman and Tversky (1979) have challenged this assumption and has shed light on the limits of this rationality. And over time, bounded rationality and cognitive biases have gained prominence in the theories that deal with human decision-making under conditions of uncertainty (Simon, 1955; Kahneman and Tversky, 1979). Along with the knowledge of bubbles in the real estate market and stocks, research has tried to explain these movements considering behavioral theories because they perceive that the decisions can vary with behavior (Steiner et al., 1998). Accordingly, the way individuals act and interpret information for investment decisions is considered in that research (Thaler, 1999).

Overall, due to the importance of the capital market for the country’s economic development and the relevant role of the analyst as a reducer of information asymmetry, it is important to study the factors that predict an analyst’s forecasting accuracy. While cognitive factors are expected to play a pivotal role in explaining an analyst’s accuracy, the contextual factors that are related to financial aspects are also likely to play a significant role. The objective of this research, therefore, was to analyze the importance of both cognitive factors (such as optimism, realism, and overconfidence) as well as financial factors (profitability, indebtedness, and volatility) in predicting the accuracy of financial analysts.

To do so, we use the Bayesian inference and classification methods, which allow expanding the research line, especially in the area of machine learning. Furthermore, the proposed training and testing methodology go beyond the validation of a model by CI, increasing the chance of success when applied in unknown cases. Hence, this research contributes to scientific advancement in the area of behavioral decision-making, not only by a joint analysis of cognitive and financial factors that influence the analyst’s forecasting accuracy but also especially the use of methodologies that consider the analysis of statistical inference and classification, both non-linear, differently from the use of traditional statistical methods used in the literature studies, such as linear regression. In addition, the research contributes to the literature that uses prediction models of analysts’ accuracy by indicating the variables that should be taken into account in these models, to increase their predictive capacity. Considering that the text of the analyst’s report is used by investors for decision-making and may influence them in this regard, the research observed cognitive biases through text analysis, which allows those interested in analysts’ reports to understand the characteristics of the disclosure. The text expresses knowledge, information, and reality created by it (Johnson and McLean, 2020), which influences its readers. The use of text analysis in this research provides evidence of the relationship between cognitive biases expressed in the text and the performance (Sydserff and Weetman, 2002) of analysts’ forecasts, whose narrative influences investor decisions, and consequently the allocation of investments in the capital market. This technique allows us to assess the main output of analysts, which is their qualitative report and is a differentiator from existing literature studies (Fogarty and Rogers, 2005).

### Analyst’s Forecast Accuracy

The accuracy of the forecast of financial analysts corresponds to how close the profit forecast disclosed by the analyst is to the actual profit obtained by the companies (Lang and Lundholm, 1996; García-Meca and Sánchez-Ballesta, 2006; Coën et al., 2009; Abernathy et al., 2013). Success in this forecast accuracy is important for the development and functioning of the capital market as it is a measure used by investors to make investment or divestment decisions, which in turn moves the capital market of a country.

Given its importance, research has identified factors that impact the analyst’s forecast accuracy, with few noting the importance of financial and cognitive aspects together. Studies that analyze financial characteristics note: (a) accounting information standards and quality (Bhat et al., 2006; Bradshaw et al., 2012; Lobo et al., 2012); (b) the analyst’s experience and skill (Brown, 1997; García-Meca and Sánchez-Ballesta, 2006; Martinez, 2007a; Kim et al., 2011); (c) quality of auditing of financial statements (Abernathy et al., 2013); (d) measurement at fair value (Ayres et al., 2017); (e) accounting standards and information quality (Tong, 2007; Xie et al., 2012); (f) corporate governance (Byard et al., 2006; Dalmácio et al., 2013); (g) company size (Lang and Lundholm, 1996; García-Meca and Sánchez-Ballesta, 2006; Ernstberger et al., 2008); (h) number of analysts who follow the company (Martinez, 2004; Ernstberger et al., 2008); (i) volatility (Lang and Lundholm, 1996; Lang et al., 2003); (j) broker size (Brown, 1997; Clement, 1999; García-Meca and Sánchez-Ballesta, 2006; Martinez, 2007a); (k) ADRs (Lang et al., 2003); (l) regulation (Kwag and Small, 2007); (m) analyst’s portfolio (Myring and Wrege, 2009); (n) team (Bandyopadhyay et al., 1995; Lys and Soo, 1995; Jaggi and Jain, 1998; Das and Saudagaram, 2002; Muslu et al., 2019).

Studies that focus on cognitive biases note: (a) selection bias (Baik, 2006, (b) overconfidence (Dittrich et al., 2005; Martinez, 2007a; Broihabnne et al., 2014; Kafayat, 2014; Lima and Almeida, 2015; Du and Budescu, 2018; Machado, 2018); (c) optimism (Easterwood and Nutt, 1999; Lim, 2001; Gervais and Odean, 2001; Ciccone, 2003; Martinez, 2007b; Corredor et al., 2014; Kafayat, 2014; Lima and Almeida, 2015; Galanti and Vaubourg, 2017); (d) anchoring bias (Brown, 1997; Marsden et al., 2008; Campbell and Sharpe, 2009; Silva Filho et al., 2018); (e) CEOs’ personal traits (Hernández-Pérez et al., 2019); (f) representativeness (Amir and Ganzach, 1998).

This article is organized as follows: the next section presents the theoretical background, followed by the methods describing the sample, operationalization of variables, and econometric models. Then, the results of this study are presented, including regression models and classification models, followed by a discussion of the results and contributions.

### Predictors of Analysts’ Accuracy

Based on the literature review, we were able to identify the main predictors of analysts’ accuracy that have been of academic interest to include both cognitive and financial, temporal, and other contextual factors, which will be described as follows.

### Cognitive Factors

In this article, we consider the most studied cognitive biases of the literature in the field of behavioral finance, related to an analyst’s accuracy. These biases are described as follows.

*Optimism* occurs when there is an overestimation of favorable performance and an underestimation of unfavorable performance (Heaton, 2002; Kafayat, 2014), or even when the individual considers that what is around him is more positive than that around other people (Harris and Hahn, 2011), or what it usually is in reality (Gervais et al., 2002). The literature states that this bias can be motivated by an intention to deceive, not to issue a report with negative forecasts, and by the fact that there is a failure in the processing of the available information (Francis, 1997). And yet, the optimistic bias in analysts may be related to conflicts of interest arising from the remuneration they receive when issuing optimistic forecasts for a company (Kothari, 2001; Martinez, 2004; Galanti and Vaubourg, 2017). Thus, this bias may be predictive of analysts’ forecast accuracy. This occurs because this bias tends to make the individual take more risks, believing they are prudent, when they are not (Kahneman, 2012), calling into question the objectivity of analysts, consequently reducing the analyst’s accuracy (Corredor et al., 2014).

*Anchoring bias* occurs when the individual makes an estimate using an initial value, adjusted to reflect new information (Tversky and Kahneman, 1974). Campbell and Sharpe (2009) highlight that this bias can lead to predictions of the results that underweight new information, which can generate forecast errors. This is because analysts may fail to properly adjust to the historical or arbitrary numbers they use as a reference (Kratz, 2016). Thus, research has considered that the anchoring effect negatively influences the forecasts of analysts (Campbell and Sharpe, 2009; Kratz, 2016; Silva Filho et al., 2018).

*Overconfidence bias* occurs when the individual overestimates his/her ability to perform a specific task (Kafayat, 2014). Although it may reinforce the characteristic of optimism (Gervais and Odean, 2001), it can also be related to a pessimistic scenario. Baumeister et al. (1989) affirm that the person with overconfidence exaggerates his/her abilities and the probability of favorable results. So, overconfidence can harm financial decisions and consequently analysts’ forecasts (Du and Budescu, 2018).

*Representativeness bias* occurs when it is considered that the occurrence of an event resembles or is representative of another so that the probability of occurrence of an event can be predicted by the occurrence of a representative event, otherwise, the probability is considered low (Tversky and Kahneman, 1974). Therefore, in the context of evaluating probabilities and predicting values, representativeness is a heuristic that leads to a series of biases and theoretical implications, whose judgment can be wrong by disregarding several other factors. In this context, Mokoteli et al. (2006) found that there is an influence of the representation bias in the analysts’ recommendations, which would explain the “irrational” behavior of these participants in the capital market. Hence, representativeness is likely to predict the analyst’s accuracy. It should be noted that Mokoteli et al. (2009) also consider that the size of the analyzed company is another potential aspect to signal the representativeness bias as analysts may assume that large companies are good and well managed to expect superior performance.

*Realism bias* occurs when there is a consideration of the individual’s experience along with reading and appreciating the events that occur in the environment (McDonald et al., 2015). More specifically, in the field of finance studies, this behavior would be related to the way that the analyst observes, interprets, and elaborates the variables about a certain company to determine the profit forecast, so realism can be predictive of accuracy (Piotr and Sina, 2015).

*Commonality* occurs when the individual considers the thought of a group or the shared experience of a group (Short and Palmer, 2008). In this sense, financial analysts tend to consider, in their forecasts, the opinions, observations, and thoughts of a group of analysts, so this can be reflected in the accuracy of their forecasts.

### Financial Factors

We have identified, through previous literature, the other non-behavioral variables that can influence the accuracy of analysts due to the context in which they are inserted that is related to financial aspects of companies, which are described in the text that follows.

*Size* is seen as the volume of a company’s assets, indicating its size in the capital market. It is understood that larger companies can present the experience and technologies that enable a better collection, forecasting, and dissemination of reliable information to the market (García-Meca and Sánchez-Ballesta, 2006; Saito et al., 2008; Ayres et al., 2017), which would influence the analyst’s forecast.

*Fair value* is representative of the use of subjective accounting practices by companies. According to the literature studies, the companies that measure assets and liabilities in this way are able to disclose their economic and financial aspects closer to reality, so that the information becomes more useful to external users (Bahadır et al., 2016), among them financial analysts, who can take advantage of the quality of greater comparability, comprehensibility, and relevance (Ayres et al., 2017) of this information, with positive reflexes in determining their forecast of results.

*The loss* represents the loss incurred by the company, which in turn is related to uncertainties (Ayres et al., 2017) and can negatively impact the profit forecast by analysts (Coën et al., 2009; Rahman et al., 2019).

*Profitability* represents the positive gain that the company was able to obtain in a given period. Therefore, it tends to be a way of motivating the company’s disclosure to the market, given the positive aspect that this is represented in terms of investment. In turn, increased disclosure provides more support for analysts to make their predictions (García-Meca et al., 2005).

*The surprise effect* occurs when there is a difference in the results between the forecast period and a previous one. So, a surprise effect represents something unexpected, which implies a scenario of greater uncertainty. Given this, although the existing literature (Abernathy et al., 2013; Magnan et al., 2015) has not directly related this variable to an analyst’s accuracy, it is reasonable to expect that there is a relationship between the variables.

*The growth* represents how much the company grew, in terms of sales, between the two periods. It is understood that companies in this condition have a greater volume of information to be considered by analysts, increasing their effort, need for time, and dedication (Barth et al., 2001), which can lead to difficulties in profit forecasts.

*Volatility* occurs when the company presents a high variation in its results. This scenario places analysts in a condition of greater uncertainty (Behn et al., 2008; Ayres et al., 2017), making it more difficult to predict their results more accurately (Saito et al., 2008).

*Indebtedness* is the state in which the company has debts with banks. These debts can increase the complexity of the analysis of a company by analysts due to the need to deal with several variables, such as interest rate, exchange rate relationship with other currencies, and the availability of credit lines (Saito et al., 2008), which may imply less precision in analysts’ forecasts (Chan et al., 1996).

*Return on Assets* (*ROA*) is a performance measure represented by the relationship between the profit that the company obtained and the need for assets to make it happen. It is also possible to expect the companies that perform better to use this condition to disseminate more information and attract the market. Then, the effect would be of a scenario that establishes better conditions for analysts to determine their forecast of results.

*Age* is the number of years in which the company issues its shares for trading on the capital market. Theoretically, companies with more years of existence have a relatively longer disclosure history and more follow-up by analysts, and, knowing the company better, the accuracy of the forecast can be more assertive (Bradshaw et al., 2012).

*American Depositary Receipts* (*ADRs*) classify the companies that trade shares in North American markets. These companies must, therefore, follow certain rules of disclosure of information that make them more transparent (Leuz, 2003), which may impact the prediction of results by analysts.

The *sector* classifies companies as a percentage of the self-regulated sectors, which are monitored by the regulatory bodies supplementary to the Securities and Exchange Commission (CVM) and can affect the quality of the disclosed information (Malaquias and Lemes, 2013). Having an effect on this quality, it can be expected that there will be an impact on the resulting forecast by an analyst.

### Time Factor

Another important factor considered in the literature for determining the analyst’s accuracy is time.

*Time* represents the interval between the analyst’s forecast and the company’s actual earnings disclosure. Prelec and Loewenstein (1991) observed that time and uncertainty are correlated such that behavioral violations occur given an inherent connection between intertemporal choices and uncertainty. In this way, a profit forecast closer to the announcement of the results can reduce the analyst’s uncertainty environment and by 5% the probability or size of the forecast error (Laverty, 1996; García-Meca and Sánchez-Ballesta, 2006).

### Other Relevant Factors

The characteristics of analysts and brokers, in addition to other contextual factors, are also considered in the literature that researches the accuracy of analysts, which will be described as follows.

*Broker size* ranks the financial analysts who work for large brokers. Jacob et al. (1999) state that the analysts of larger brokers have access to more resources and more sophisticated forecasting models, which contribute to the quality of their forecasts.

The company’s *popularity* is noted by the number of analysts who follow the company. Although a few studies considering a direct relationship of this variable with the accuracy of analysts have not been identified in the literature, it is expected that companies followed by a larger number of analysts present greater support for the analysts themselves, contributing to the accuracy of their forecasts.

*Analyst specialization* is identified by the smallest number of sectors followed by a financial analyst. Bolliger (2004) considers that the analyst’s specialization can reduce the complexity of his work by gaining similarity.

*Analyst experience* in a given company is perceived by the volume of income forecast that the analyst performed for a given company. It also seems to make sense to consider that the greater the analyst’s experience in a given company, the greater his knowledge of it, and the greater the chance of accuracy in his forecasts.

*Experience* represents the time spent by an analyst in preparing and issuing profit forecasts. Martinez (2004) considers that the time that the analyst acts in this function determines a greater experience, which in turn can be beneficial in terms of accuracy of the profit forecast, but that, according to García-Meca and Sánchez-Ballesta (2006), the greater the number of companies followed by the analyst, the more extensive is their portfolio, which can negatively impact the accuracy of their forecast.

The analyst *portfolio* represents the number of companies followed by the analyst. García-Meca and Sánchez-Ballesta (2006) consider that a greater number of companies followed by a given analyst is called the analyst’s portfolio complexity, which can lead to a greater need for the analyst to dedicate time to companies, affecting their individual attention to each of them. Therefore, negatively affecting analysts’ forecasts (Ledbetter et al., 2014; Wang et al., 2019).

**FIGURE 1:**
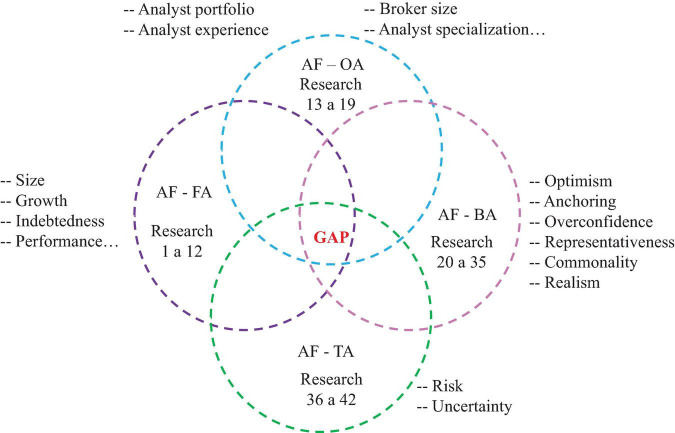
Research intersection. AF, analyst forecast; OA, others aspects; AF, financial aspects; BA, behavioral aspects; TA, temporal aspects.

A summary of the main themes related to the proposed study and the area of the research gap can be seen in the Appendix. The intersection of the subjects to be studied can be seen in Figure 1.

## Materials and Methods

### Study Delimitation and Methods

In 2019, the study was conducted in publicly traded Brazilian companies. To compile the database, financial information was collected from the S&P Capital and Thomson Reuters^®^ databases, and the analysts’ reports were obtained *via* the Thomson One^®^ systems, which were used to collect the estimated earnings per share, the estimated date, the name of the broker, and the analyst. Initially, as shown in Table 1, the sample started with 338 Brazilian companies, reaching a final sample of 94 companies. As the reports by analysts and companies were collected quarterly, the database ended with 1,026 observations. Diction^®^ software was used to read the content of the analysts’ reports and calculation of some cognitive biases.

**TABLE 1:** Definition of the final sample in 2019.

Procedures for selecting the final sample	Brazil
Initial sample	338
(–) Financial	34
(–) Without estimated LPA	181
(–) Lack of accounting data	29
**(=) Final number of companies**	**94**

### Definition of Variables and Econometric Model

In terms of methodology, this research analyzed the relationship of the variables with the accuracy of analysts in the way it was generated, that is, as a variable of the reason type, continuous. The accuracy of the analyst’s forecast was calculated based on previous research (Coën et al., 2009; Abernathy et al., 2013), according to Equation (1):


(1) $$AC=1-\left|\left(\frac{EPS_{real}-EPS_{prev}}{EPS_{real}}\right)\right|$$

where:

EPS_real_ = corresponds to realized earnings per share and

EPS_prev_ = corresponds to the predicted earnings per share, based on the analysts’ consensus (average).

Equation (1) calculates the analyst’s accuracy as the difference between the firm’s actual earnings per share and the analyst’s estimated earnings per share, weighted by the firm’s actual earnings per share. “AC” represents the dependent variable in the study.

During that time, two regression models were considered: (a) a model with the assumption of linearity between the relationships, through Ordinary Least Squares (OLS) and (b) a non-parametric model *via* Random Forest (RF). In the second step, the accuracy variable was categorized to allow analysis from the point of view of classification models, applied in two ways: the Bayesian model and RF. In terms of categorization, the accuracy was separated into the following:

(a) terrible, for negative values;
(b) median, in the case of values between 0 and 0.6;
(c) good, for values between 0.6 and 0.85; and
(d) high, in the case of values above 0.85.

That is, categorical accuracy (AC) was defined as:


AC=terribleAC<0.0;median  0.0≤AC≤0.6;

(2) good  0.6<AC≤0.85;highAC>0.85

So far, no studies were found that used the analysts’ categorization of accuracy to be used as a parameter. Thus, to categorize this variable now in this study, we used percentile measures of the distribution of analysts’ accuracy values. We took care not to create a high number and categories, which could cause tables with values equal to 0 in some subcategories. This categorization also considered the representativeness of the accuracy values. For example, in the case of a negative value for the analysts’ accuracy, it was considered that there was a representative error in their forecasts, which were classified as “terrible,” as we considered them highly undesirable for the capital market.

As the method provides categorization analysis, a cross-tabulation without variable data could impair these analyses. The variables used to model the accuracy of the analyst’s forecast are grouped, and cognitive, financial, temporal, and other aspects are described as follows:

Fact = Variables representing cognitive and temporal factors, being used here:

(a) *Optimism or Optim*: a variable representing an optimistic profit forecast made by the analyst for a given company, being 1 when the analyst’s forecast extrapolates the consensus of other analysts who issued profit forecasts for the same company (Corredor et al., 2014; Galanti and Vaubourg, 2017).
(b) *Anchoring or Ancho*: a dummy variable representing the anchoring effect, being 1 (one) if the analyst’s forecast is between the real earnings per share and the anchor, 0 (zero) the opposite. For this study, the real earnings per share in *t* − 1 were used as an anchor.
(c) *Overconfidence or Overconf*: a variable representing the analyst’s overconfidence obtained through the software Diction^®^, which considers the language used in the analysts’ reports, more specifically the use of terms that indicate trust, such as “always,” “totally,” “absolute,” etc., reduced to the terms of hesitation, such as “perhaps” and “supposedly.”
(d) *Representativeness or Repres*: a variable related to the analyst’s representativeness aspect, calculated by analyzing the text of the analysts’ report, *via* Diction^®^, which considers expressions such as “challenging,” “dominated,” “motivated,” “influencing,” etc., reducing terms such as “examine,” “reasonable,” and “indifferent.”
(e) *Realism or Real*: a variable related to the aspect of realism in the language of the analysts’ report, obtained *via* Diction^®^, which considers the terms that describe tangible, immediate, and recognizable issues affecting people’s daily lives, such as “local,” “municipality,” “instant,” “obsolete,” etc. and disregarding others related to the past or abstract.
(f) *Commonality or Common*: a variable representing a text focused on centrality and cooperation, obtained *via* Diction^®^, considering terms such as “standardized,” “conformity,” “alignment,” and “equivalent,” etc. and disregarding the other ones that represent diversity and exclusion such as “inconsistent,” “extremist,” “self-sufficient,” etc.
(g) *Time*: a variable representing the interval between the date on which the forecast was made and the date of the earnings release, considering here the end of 2019;

Size = a variable representing the size of the company, calculated by logging the value of Total Assets. A positive relationship with accuracy is expected;
Fair Value or FV = dummy, indicating 1 (one) if the company has assets or liabilities at a fair value, 0 (zero) otherwise;
Loss = dummy, with 1 (one) if the company has a loss, 0 (zero) the opposite;
Profitability or Profit = the company’s profitability, calculated by the ratio between Ebitda and Total Assets;
Surprise or Supr = company surprise, estimated through the ratio between a variation in the profit between the two periods and the profit in *t* − 1;
Growth = company growth, as measured by a variation in sales revenue;
Volatility or Volat = volatility of the company’s results, estimated by the logarithm of the ratio between the SD of profit from the previous five quarters and the average profit module;
Indebtedness or Indeb = corporate indebtedness, calculated by the ratio between total liabilities and total assets;
Performance or ROA = a variable representing the company’s performance, estimated by the ratio of net profit and total assets;
Age = age of the company, given by the difference between the year of opening of the company and the year of 2019;
American Depositary Receipts or ADR = dummy, with 1 (one) for the companies that issue ADRs, 0 (zero) the opposite;
Sector = dummy, being 1 (one) for companies belonging to the self-regulated sector, 0 (zero) the opposite;
Broker Size or SizeBrok = a variable representing the size of the broker and calculated according to the number of companies followed by the broker;
Popularity or Popul = a variable representing the company’s popularity and calculated by the number of analysts who follow the company;
Analyst’s specialization or SpecAna = a variable that represents the analyst’s specialization and is found by the number of sectors followed by the analyst;
Analyst’s experience or ExperAna = a variable that represents the analyst’s experience in a given company and is calculated by the analyst’s forecast volume in a given company;
Analyst’s experience or Exper = a variable that represents the analyst’s experience in this function and is determined by the number of forecasts in 2019;
Portfolio or Portf = a variable that represents the complexity of the analyst’s portfolio and is calculated by the number of companies followed by him.

From these variables, regression models were developed, in which the accuracy defined in Equation (1) is the independent variable, and classification models, in which the response variable was obtained from the categorization of accuracy. The regression models studied were Multiple Linear Regression (MRL) with the OLS method and the non-parametric regression method—RF Regression. In the case of MRL, the OLS econometric model has the general shape described by:


(3) $$\begin{array}{c} {\text{AC}}_{i,t}=\alpha_{0}+\beta_{1}{\text{Fact}}_{i,t}+\beta_{2}{\text{Size}}_{i,t-1}+\beta_{3}{\text{FV}}_{i,t-1}+\beta_{4}{\text{Loss}}_{i,t-1}\\ +\beta_{5}{\text{Profit}}_{i,t-1}+\beta_{6}{\text{Surp}}_{i,t}+\beta_{7}{\text{Growth}}_{i,t-1}+\beta_{8}{\text{Volat}}_{i,t-1}\\ +\beta_{9}{\text{Indeb}}_{i,t-1}+\beta_{10}{\text{Roa}}_{i,t-1}+\beta_{11}{\text{Age}}_{i,t}+\beta_{12}{\text{ADR}}_{i,t}\\ +\beta_{13}{\text{Sector}}_{i,t}+\beta_{14}{\text{SizeBrok}}_{i,t}+\beta_{15}{\text{Popul}}_{i,t}+\beta_{16}{\text{SpecAna}}_{i,t}\\ +\beta_{17}{\text{ExperAna}}_{i,t}+\beta_{18}{\text{Exper}}_{i,t}+\beta_{19}{\text{Portf}}_{i,t}+\varepsilon_{it} \end{array}$$

where AC = analyst’s forecast accuracy that is calculated according to Equation (1), β*_*i*_* are the coefficients to be determined by the OLS method. The non-parametric RF model does not have an analytical expression but does provide the importance of predictive covariates in the form of ranking.

The RF method, based on an RF algorithm, is a machine learning technique that has been used in general in psychology research. This method is a classifier based on decision trees, which can be used in regression or classification problems (Fürer et al., 2020), as used in this study.

To analyze the correlation between continuous variables, Spearman’s correlation test was applied, and the test results can be seen in Table 2.

**TABLE 2:** Spearman’s correlation.

	Ac	Overconf	Repres	Real	Common	Time	Size	Profit	Surp	Growth	Volat	Indeb	ROA	Age	Popul	SpecAna	ExperAna	Exper
Overconf	0.0364																	
Repres	–0.0306	0.4069***																
Real	–0.0450	–0.0102	0.0150															
Common	0.0454**	0.0464	–0.0078	–0.0477														
Time	−0.0707**	–0.0206	0.0225	0.0374	−0.0698**													
Size	0.0409	0.0402	–0.0316	–0.0313	−0.1141***	–0.0009												
Profit	0.2032***	0.0370	–0.0108	0.0693**	0.0044	0.0421	–0.0259											
Surp	0.4125***	–0.0241	–0.0104	0.0027	0.0587*	0.0071	0.1192***	0.1451**										
Growth	0.0241**	−0.0633**	−0.0623**	0.0451	0.0207	0.0016	–0.0164	0.0693**	0.3043***									
Volat	0.0062	0.0227	–0.0441	–0.0103	0.0501	–0.0248	0.2050***	−0.0525*	−0.1219***	–0.0318								
Indeb	−0.1504***	0.0548*	0.0079	0.0099	–0.0508	0.0059	0.2907***	−0.1189***	−0.1932***	−0.0634**	0.5660***							
ROA	0.2038***	−0.0773**	−0.0761**	0.0296	0.0306	0.1281***	–0.0464	0.4657***	0.2452***	0.0237	−0.2947***	−0.2829***						
Age	−0.0490**	0.0353	–0.0483	–0.0061	–0.0006	0.0596*	0.2130***	−0.0895***	0.0392	–0.0381	0.1640***	0.1323***	0.0836**					
Popul	−0.0888***	0.0060	0.0054	–0.0387	−0.1443***	0.0322	0.5890***	0.0833***	−0.0589*	−0.1168***	−0.1460***	0.0506	0.1361**	0.1840***				
SpecAna	0.0480*	–0.0127	–0.0192	0.0121	0.2250***	0.0268	−0.3645**	0.0160	–0.0082	0.0922***	0.1040***	–0.0018	–0.0258	0.0676**	−0.3936***			
ExperAna	0.0278	0.0203	0.0230	0.0610*	0.0498	−0.0788**	0.1526***	−0.1152***	0.0473	–0.0011	0.0692**	0.1244***	−0.0738**	0.0589*	0.0450	0.0640**		
Exper	0.0610***	–0.0336	–0.0337	0.0804**	0.2475***	–0.0207	−0.1852**	–0.0254	–0.0341	0.0466	0.1709***	0.0259	0.0263	0.0432	−0.4011***	0.6956***	0.3723***	
Portf	0.0729***	–0.0388	–0.0423	0.0614**	0.2171***	0.0141	−0.3296**	0.0181	–0.0312	0.0839***	0.1021***	–0.0450	0.0443	–0.0328	−0.4946***	0.7927***	0.0996***	0.8893***

Being: *, **, *** significant at 1%, 5% and 10%, respectively.

The results indicated that the main observed correlations occur between the variables Size and Popul, SpecAna, and Portf; Profit and ROA; Popul and Expert and Port; SpecAna and Expert and Portf. Despite these correlations, the VIF test showed a result of 1.59, which indicated that multicollinearity is not a concern. Furthermore, the article evolves in the methods, applying the RF model which, as it is non-parametric, the interdependencies of the variables are unrestricted. Therefore, the greatest advantage of RF models is that it is insensitive to multicollinearity (Imel et al., 2015).

## Results

### Regression Models

In the models presented in this section, the accuracy of the analyst’s forecast was considered as described in Equation (1) being a reason-type variable. Initially, the OLS regression was run, assuming a linear relationship between the variables, the results of which are found in Tables 3, 4. OLS was applied with the Caret package (Kuhn, 2016), which allows for cross-validation, in that the model is obtained and validated through training samples. Resampling in training avoids overfitting, which would lead to high values for betas and models with high variance (a good prediction in the training sample and a weak prediction in the test sample). To fit the model, a training sample was defined with 70% of the observations, reserving 30% of the data to verify the accuracy of the model in the test sample. The test and training samples were obtained randomly.

**TABLE 3:** Regression in Ordinary Least Squares (OLS), carried out in the Caret package, for 2019.

	General		Otim		Ancho		Overconf	
	Coef.	T	Coef.	t	Coef.	t	Coef.	t
**Otim**	–0.86	−2.304**	–1.08	−3.001***				
**Anco**	0.84	1.997**			1.17	2.919***		
**Overconf**	–0.02	–0.924					–0.03	–1.16
**Repres**	–0.06	–0.572						
**Real**	–0.02	–0.274						
**Common**	0.07	1.11						
**Time**	0.00	–0.819						
**Size**	–0.11	–0.18	0.26	0.43	–0.09	–0.14	0.22	0.36
**FV**	1.69	2.355**	1.45	2.029**	1.58	2.204**	1.42	1.974**
**Loss**	–0.84	–1.02	–0.85	–1.044	–0.83	–1.02	–0.86	–1.05
**Profit**	34.92	3.096***	34.85	3.106***	38.13	3.414***	39.03	3.470***
**Surp**	0.00	–0.348	–0.01	–0.43	–0.01	–0.45	–0.01	–0.63
**Growth**	–0.99	–0.952	–1.18	–1.133	–0.73	–0.70	–1.03	–0.99
**Volat**	0.13	1.60	0.13	1.667*	0.13	1.58	0.14	1.764**
**Indeb**	–5.77	−6.031***	–5.83	−6.140***	–5.68	−5.956***	–5.96	−6.245***
**ROA**	–28.53	−1.943*	–26.65	−1.833*	–29.47	−2.031**	–30.99	−2.118**
**Age**	–0.01	−1.798*	–0.01	−1.845*	–0.01	–1.72	–0.01	–1.65
**ADR**	0.62	1.39	0.81	1.849*	0.59	1.32	0.84	1.906*
**Sector**	0.59	1.06	0.47	0.85	0.77	1.39	0.61	1.10
**SizeBrok**	0.29	0.658	0.23	0.53	0.24	0.55	0.18	0.42
**Popul**	–0.05	–0.43	–0.13	–1.22	–0.05	–0.42	–0.13	–1.25
**SpecAna**	0.13	0.807	0.15	0.93	0.16	1.02	0.16	1.02
**ExperAna**	–0.06	–0.334	–0.04	–0.26	–0.06	–0.34	–0.03	–0.20
**Exper**	0.07	1.33	0.07	1.44	0.07	1.37	0.07	1.42
**Portf**	–0.23	–1.45	–0.25	–1.60	–0.23	–1.50	–0.25	–1.59
**Constant**	3.51	0.647	1.82	0.98	1.28	0.69	2.60	1.23
***R*^2^-training**	0.1527		0.1398		0.1467		0.1274	
***R*^2^-test**	0.1755		0.1509		0.1679		0.1379	
**Error-training**	3.8849		3.8850		3.8806		3.9053	
**Error-test**	2.5818		2.6312		2.5987		2.6539	

Being: *, **, *** significant at 1%, 5% and 10%, respectively.

**TABLE 4:** Regression in OLS, carried out in the Caret package, for 2019.

	Repres		Real		Common		Time	
	Coef.	*T*	Coef.	*t*	Coef.	*t*	Coef.	*t*
**Repres**	–0.12	–1.23						
**Real**			0.05	1.25				
**Common**					0.06	1.680*		
**Temp**							–0.002	–1.30
**Size**	0.18	0.31	0.11	0.19	0.08	0.13	0.21	034
**FV**	1.39	1.936*	1.50	2.081**	1.52	2.108**	1.42	1.982**
**Loss**	–0.99	–1.21	–0.95	–1.16	–0.93	–1.14	–0.83	–1.01
**Profit**	37.55	3.342***	37.67	3.354***	37.79	3.369***	37.06	3.292**
**Surp**	–0.01	–0.58	–0.01	–0.55	–0.01	–0.57	–0.01	–0.49
**Growth**	–0.96	–0.92	–1.04	–0.99	–1.04	–1.00	–0.97	–0.93
**Volat**	0.14	1.768*	0.14	1.740*	0.14	1.724*	0.13	1.679*
**Indeb**	–5.90	−6.188***	–6.04	−6.307***	–6.08	−6.352***	–5.86	−6.133***
**ROA**	–3.12	−2.132**	–3.01	−2.064**	–3.05	−2.090**	–2.74	−1.863*
**Age**	–0.01	−1.742*	–0.01	−1.843*	–0.01	−1.830*	–0.01	−1.728*
**ADR**	0.81	1.849*	0.79	1.802*	0.77	1.768*	0.79	1.814*
**Sector**	0.61	1.09	0.51	0.92	0.49	0.89	0.59	1.06
**SizeBrok**	0.15	0.35	0.17	0.39	0.14	0.33	0.10	0.24
**Popul**	–0.13	–1.23	–0.12	–1.12	–0.11	–1.06	–0.13	–1.20
**SpecAna**	0.16	0.99	0.14	0.91	0.13	0.80	0.16	1.02
**ExperAna**	–0.04	–0.25	–0.03	–0.20	–0.04	–0.25	–0.05	–0.29
**Exper**	0.07	1.46	0.07	1.41	0.07	1.44	0.07	1.40
**Portf**	–0.25	–1.63	–0.24	–1.55	–0.24	–1.53	–0.24	–1.53
**Constant**	7.32	1.43	–0.26	–0.11	–0.97	–0.41	1.84	0.97
***R*^2^-training**	0.1213		0.1348		0.1348		0.1336	
***R*^2^-test**	0.1380		0.1444		0.1495		0.1546	
**Error-training**	3.9189		3.8948		3.8940		3.8959	
**Error-test**	2.6475		2.6381		2.6262		2.6146	

Being: *, **, *** significant at 1%, 5% and 10%, respectively.

The training sample is inserted in the RF algorithm, after being partitioned with cross-validation, *via* the Caret package, considering *k*-fold equal to 10, that is, 10 unfolding or random drawing, which was enough to calibrate the model, achieving stability. This means that, through cross-validation, 10 models are created, by drawing 10 different samples from the one used for training. This process of finding the model and applying it multiple times allows us to arrive at a more tuned model, which in turn is applied to the test model.

Primarily, the objective of the research was a joint analysis of cognitive and financial factors influencing the accuracy of the analysts’ profit forecast, which can be seen in the results of the General column, in Table 3. In this sense, the OLS analysis shows that cognitive biases such as optimism and anchoring are related to the accuracy of the analyst’s forecast. As for financial factors, fair value, profitability, indebtedness, performance, and age were also related to the accuracy of the analyst’s forecast. To observe the effects of cognitive and temporal aspects in isolation, it was decided to analyze these aspects separately. As a result, it was confirmed that optimism and anchoring are related to the analyst’s accuracy. With regard to optimism, an indication of the negative relationship with accuracy is in line with what was expected by the literature (Kothari, 2001; Martinez, 2004; Galanti and Vaubourg, 2017), but a positive result obtained with anchoring differs from what was expected (Campbell and Sharpe, 2009; Kratz, 2016). Additionally, commonality was also positively related to the accuracy, as expected by the literature, with emphasis on the use of shared experiences (Short and Palmer, 2008).

**FIGURE 2:**
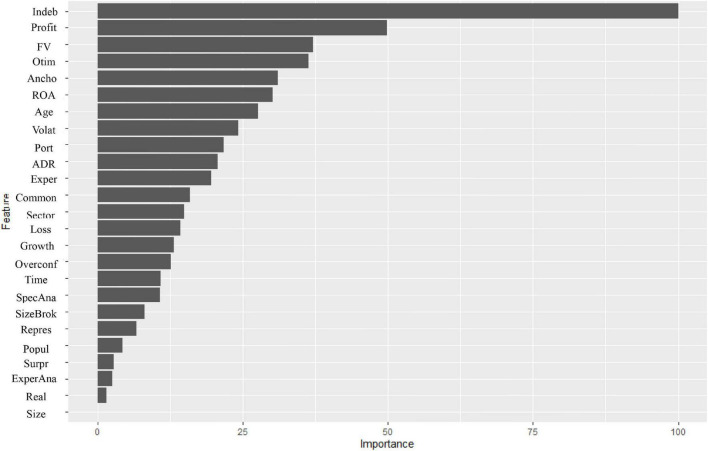
Importance of variables in Ordinary Least Squares (OLS).

Additionally, we can observe, through the linear method of regression in OLS, signaling of the most important factors to explain the analyst’s accuracy, as shown in Figure 2. It is possible to observe that indebtedness, profitability, and fair value stand out as financial aspects that interfere with the accuracy of the analyst, and that optimism and anchoring also have an importance in determining accuracy, and, to a lesser extent, commonality.

Based on the results in OLS, the explanatory power is low (*R*^2^ values around 0.14), however, there is an alignment in the explanatory power of the training and test models, showing consistency between the models generated. However, some variables can influence accuracy in a non-linear way. We emphasize that this result of the value of *R*^2^ can be explained by considering the linearity of the applied model, so that, next, we propose to explore the use of non-linear models, which helped to improve the value of *R*^2^. Thus, to observe the factors that relate to the analyst’s accuracy under this prerogative, the study analyzed the relationships through a non-parametric model, the RF model (RF Regression), and the results of which are presented in Table 5.

**TABLE 5:** Regression in Random Forest (RF) in 2019.

	General	Otim	Ancho	Overnconf	Repres	Real	Common	Time
**Error – training**	2.1240	2.0907	2.2585	2.2739	2.2611	2.2835	2.2800	2.2420
***R*^2^-training**	0.6965	0.6986	0.6737	0.6579	0.6627	0.6533	0.6544	0.6711
**Trees**	13	10	10	10	10	10	10	10
**Error – test**	1.8320	1.7953	2.1536	2.1601	2.1584	2.2154	2.1202	2.1314
***R*^2^-test**	0.5772	0.5928	0.5005	0.4867	0.4878	0.4812	0.5045	0.5055

In the general model, the best explanatory power was achieved using the arrangement of 13 trees, while in the individual models, the arrangement indicated was 10 trees. With regard to the OLS analysis, the explanatory power of the models increased. We observe the *R*^2^ (around 67% in the training sample and 51% in the test sample). Thus, although the variance for the RF model is greater than the variance for the OLS model, there are indications of non-linear effects between the variables considered as the RF had an average increase of 53% in the explanation of the training sample and an average increase of 36% in the test sample when compared to the OLS model.

**FIGURE 3:**
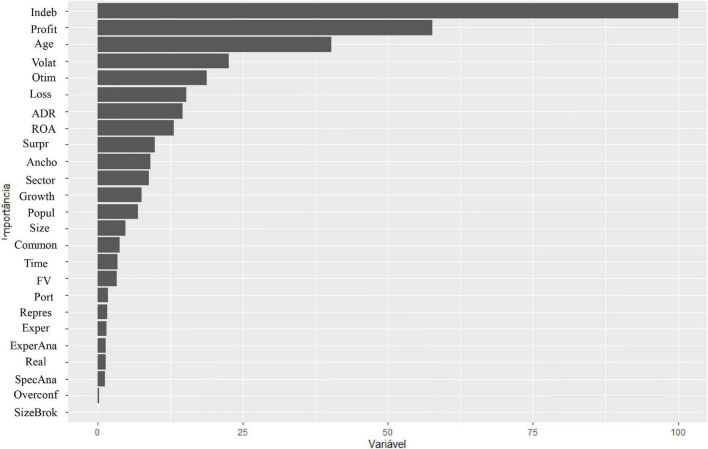
Importance of variables in Random Forest (RF).

In comparison with the result in OLS, Figure 3 shows, when considering a non-parametric model, that, in fact, indebtedness, profitability, and optimism are the factors of the greatest importance for the accuracy of the analysts’ profit forecast but highlights age and volatility as factors greater than fair value and anchoring, which were evidenced by the OLS method. Therefore, these variables, although not significant in linear regression, seem to have some importance in determining the analyst’s accuracy.

### Classification Models

**FIGURE 4:**
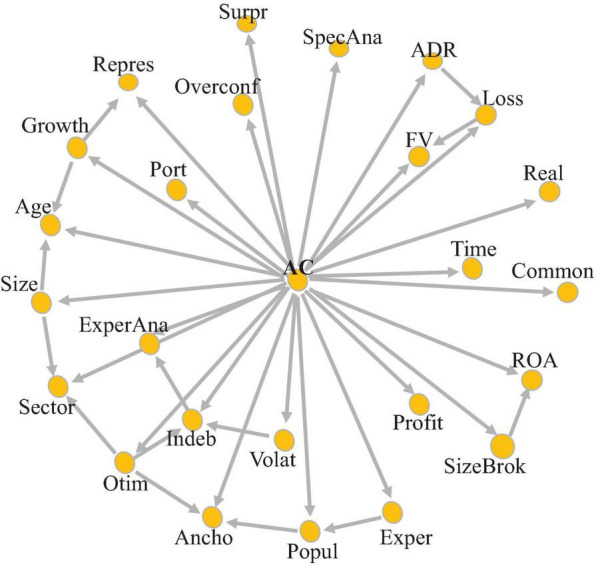
List of variables in the *k*-dependence Bayesian (*k*-DB) classification model, with a value of *k* = 5.

In the models presented in this section, accuracy (AC) was categorized according to the criterion informed in Expression (2). Two classification models were considered: the Bayesian Network model and the RF model. Training samples (70% of the data) and test samples (30% of the data) were also used. Regarding the classification technique by means of the Bayesian Network, Hill-climbing *k*-dependence Bayesian classifier (*k*-DB) was used through the bnclassify package (Mihaljević et al., 2018) of software R. The bnclassify package uses only discrete variables, thus each variable presented was categorized. The first step consists of learning the network structure, which contains the relationships between the variables. As a result of this step, by applying the *k*-DB model in up to 5 generations (*k* = 5), the network structure was obtained (Figure 4).

The graph illustrates an interdependence characteristic between the variables considered. What is perceived is that there are variables that are not interrelated, such as overconfidence, commonality, realism, surprise, analyst specialization, time, profitability, and analyst portfolio. This means that they are shown to be disconnected from the others, while there is a network of relationships between the other variables. For example, accuracy is influenced by volatility and indebtedness, but indebtedness and volatility are related. Thus, given that the estimate for the analyst’s accuracy variable is written as a product of conditional probabilities, the change that occurs in some variables is propagated by the network, and directly affects the analyst’s accuracy, through the variable Indeb, or indirectly, due to the impact that the variable Indeb causes on the variable Volat, which, in turn, affects the accuracy of the analyst.

Therefore, as the results of the RF models showed a greater explanatory power due to the non-linearity of the variables, the classification model by Bayesian networks allows us to verify which are the closest neighbor variables that alter a variable. We highlight some groups in this regard, such as the interrelationship between ADR, Loss, and FV, a point that shows that the anchor used by the analyzer has an influence of its optimistic bias and the company’s popularity, while the probability of optimistic behavior is influenced by the indebtedness of the company. These results corroborate the RF model.

From the network structure, the conditional probability tables were obtained and the model was calibrated. The results of these classifications are shown in Table 6.

**TABLE 6:** Confusion matrix for the *k*-dependence Bayesian (*k*-DB) classification model in 2019.

		Reference – training					Reference – test		
	
Prediction	Poor	Median	Good	High	Prediction	Poor	Median	Good	High
Poor	189	18	12	2	Poor	82	14	5	4
Median	8	92	14	11	Median	4	36	6	1
Good	12	17	124	23	Good	7	6	34	20
High	7	5	26	151	High	1	3	23	61
Accuracy	0.7744				Accuracy	0.6938			
Kappa	0.6956				Kappa	0.5847			

Through the results presented in Table 6, there is a good adjustment of the classification model. In training, the accuracy of the model was greater than 77%, remaining around 70% when applied to the test sample. This satisfactory adjustment can also be observed diagonally in the relationship between the reference and prediction values, indicating the model’s correctness.

Finally, it was still possible to apply the non-parametric RF classification technique, *via* the Caret package (Kuhn, 2016). It is noteworthy that the *k*-DB model analyzes indirect effects propagated in the network, while the RF model is of the “decision tree” type, which tries to provide more information based on the data partition. The results are shown in Table 7 (confusion matrices) and Table 8 (importance of the variables).

**TABLE 7:** Rating Model in RF in 2019.

		Reference – training					Reference – test		
	
Prediction	Poor	Median	Good	High	Prediction	Poor	Median	Good	High
Poor	82	14	5	4	Poor	82	14	5	4
Median	4	36	6	1	Median	4	36	6	1
Good	7	6	34	20	Good	7	6	34	20
High	1	3	23	61	High	1	3	23	61
Accuracy	0.6938				Accuracy	0.6938			
Kappa	0.5847				Kappa	0.5847			

**TABLE 8:** Importance of variables using the RF classification model in 2019.

	Importance		Importance		Importance
Volat.c.Q	100	Size.c.L	5.957	Port.c.C	1.39
Surp.c.L	65.127	Surp.c.C	5.845	Exper.c.Q	1.376
Surp.c.Q	58.25	Age.c.L	5.64	Real.c.C	1.265
Loss1	45.761	Indeb.c.L	5.114	Sizeroker1	1.257
Volat.c.L	45.313	Size.c.C	5.078	Especia.c.L	1.255
Profit.c.L	40.512	ADR1	4.781	Real.c.Q	1.027
Otim1	34.861	Volat.c.C	4516	Especia.c.C	1.024
Sector1	33.513	Growth.c.C	4.417	Overnconf.c.Q	0.827
Anco1	28.636	Popul.c.Q	4.041	Experi.c.L	0.786
Profit.c.Q	23.082	ROA.c.L	3.919	Experi.c.Q	0.731
Popul.c.C	22.743	FV1	3.73	Common.c.L	0.652
Growth.c.L	19.636	Port.c.L	3.128	Time.c.C	0.64
Indeb.c.Q	19.061	ROA.c.C	2.621	Overnconf.c.L	0.601
Popul.c.L	17.777	Port.c.Q	2.58	Repres.c.C	0.465
Size.c.Q	12.206	Growth.c.Q	2.575	Exper.c.C	0.44
Indeb.c.C	11.694	Time.c.L	2.003	Common.c.Q	0.403
Profit.c.C	11.299	Specia.c.Q	1.964	Real.c.L	0.347
ROA.c.Q	7.447	Exper.c.L	1.451	Repres.c.Q	0.292
Age.c.Q	6.632	Experi.c.C	1.416	Repres.c.L	0.241
Age c.C	6.389	Time.c.Q	1.398	Overnconf.c.C	0.09

*C, median; Q, good; L, high.*

In Table 7, it is possible to observe that the RF classification model also presented a satisfactory adjustment for training (accuracy of more than 85) and for the test (accuracy of the model above 70%), which is also observed in the diagonal of the relationship between the reference and prediction values. One of the advantages of using RF through the Caret package is the possibility of obtaining the list of variables with their respective importance, in which case the importance of the categories of variables is evaluated. In this RF classification model, profitability, optimism, and volatility are the aspects that are more related to the accuracy, similar to the results found in the regression models, both linear and non-linear. However, it highlights the surprise and the loss, which did not appear in the regression models as significant.

Therefore, this study indicates that the most important factors in the analyst’s accuracy, in financial terms, are indebtedness, profitability and volatility, and, in cognitive terms, optimism. However, other aspects, such as the cognitive factors of anchoring and commonality, as well the financial factors of the use of fair value, age, surprise in the results, and even the issuance of ADRs, also have relevance but to a lesser degree.

## Discussion

The results of this study provided evidence of the influence of both cognitive and financial factors in the profit forecast accuracy of financial analysts. Among the cognitive factors, biases of optimism, anchoring, and commonality had an impact on the analysts’ accuracy. Surprisingly, realism, overconfidence, and representation had no impact. The time factor of the estimate itself did not have an impact either.

More specifically, we found a negative relationship between optimism and accuracy. A previous literature study states that the optimistic bias of analysts may occur due to the existence of a conflict of interest, which may occur for reasons such as real intention to deceive; selection by deciding not to report under negative forecast conditions; improperly processing available information; and for the remuneration, they receive (Francis, 1997; Kothari, 2001; Martinez, 2004; Galanti and Vaubourg, 2017). Added to this is the fact that, in Brazil, we have a less developed capital market, in addition to less stable macroeconomic and political environments (Hillier and Loncan, 2019; Duran and Stephen, 2020), which may justify the negative relationship found as such a scenario adheres to a higher level of uncertainty for analysts (Pastor and Veronesi, 2013) who, when issuing optimistic forecasts, may incur a greater error.

Furthermore, the results indicated that anchoring impacts the accuracy of analysts in a positive way. This result goes against the idea that has been put forward in the literature (Tversky and Kahneman, 1974), which considers that the use of an initial number to make an estimate tends to have an insufficient adjustment, causing the so-called anchoring bias. A previous literature study considers that these failures can interfere with the proper use of historical or arbitrary numbers that analysts use as a reference (Kratz, 2016), to expect a negative relationship with accuracy (Campbell and Sharpe, 2009; Kratz, 2016; Silva Filho et al., 2018). But, we found the relationship in the opposite direction, thus, opening avenues for future research to further understand the impact of anchoring on analysts’ profit forecasts.

The result with respect to commonality is highlighted, which points to a positive effect of this bias on the analyst’s accuracy, following what was expected in the literature, as the text centered on considering the opinion of the community, of other groups, can lead to greater sharing of values, meanings (Etzioni, 2001; Wong Un, 2002; Brah, 2006; Short and Palmer, 2008), leading to greater accuracy of analysts. On the other hand, unlike idiosyncratic behavior, characterized by the emotional distance of belonging groups and the primacy of personal goals in relation to group goals, allocentric behavior values integrity and solidarity ties to the belonging groups, conditioning their self-concepts and behaviors to these groups and perceive them as harmonious, hierarchical, and homogeneous, in addition to being eminently distinct from the other groups (Ferreira et al., 2002). In this way, when collectivism prevails, it is more likely to be influenced by belonging groups. Therefore, a relationship is created between communal behavior and the improvement of the financial analyst’s informational environment. By taking into account the opinion of other members, the analyst has access to a greater volume of information, thus facilitating his forecasting work.

Among financial factors, we found that fair value, profitability, ROA had a positive relationship with analyst accuracy while indebtedness and company age had a negative relationship. Additionally, classification tests showed that indebtedness, profitability, and volatility had a strong impact. Fair value in company accounting, age, performance, surprise results, disclosure of losses, and underwriting of ADRs are also of importance. On the other hand, aspects that do not seem to matter in terms of the analysts’ accuracy were the size of the company, the analyst’s experience and expertise, and the company’s popularity.

In addition to contributing to a more comprehensive understanding of the analyst’s forecast accuracy by investigating both cognitive and financial factors, this research brings directly an important result for scientific research in the finance area, by noticing that accounting information at fair value positively and strongly affects the accuracy of analysts, and with outstanding importance. This result confirms that fair value information, sometimes subjective and observed in the context of information management, allows greater comparability between companies, more clearly reflects current economic effects and administrative decisions, and makes information more understandable and relevant for analysts, corroborating the studies of Carroll et al. (2003); Milburn (2008), and Brînză and Bengescu (2016), and contrary to the idea that the possible increase in the volatility of results would compromise the analyst’s accuracy, as observed in other studies (Barth and Taylor, 2010; Riedl and Serafeim, 2011; Magnan et al., 2015; Ding et al., 2017). Thus, the indications are that the benefits outweigh the costs of this accounting practice, so this research helps to contribute to this literature.

A limitation of the study was that the sample was limited to 1 year. Further, the data were obtained from Brazilian publicly traded firms. Future research from other countries, especially with regulatory differences, varied capital market development, investor protection, and quality of accounting information, could verify whether the most important variables for the determination of analysts’ accuracy would be different. By considering different countries, studies can expand the consideration of economic variables and better control the heterogeneity of omitted factors. Finally, it should be noted that choosing the categorization of the accuracy variable can imply changes in the result. Although the tests did not indicate significant differences, it is important that the researchers are aware of this and new tests are carried out in future research. Further research can also vary the way of observing cognitive variables and also explore new statistical learning techniques for analysis.

### Contributions and Future Research

The research differential lies in the observation of cognitive aspects through text analysis, the identification of the importance of cognitive biases in the analyst’s accuracy so far not identified in other studies, the joint analysis of cognitive and financial factors, and the perspective of a methodological advance, when comparing causal dependency models (OLS) with probabilistic dependency models. Furthermore, the study brings to future research other contributions of a technical and practical nature for analysts, brokers, and investors by identifying the most important factors for accuracy in forecasting models, through the use of RF.

As a contribution to existing research, the study reaffirms that cognitive biases are capable of influencing decisions and makes more evident the importance of these biases for the accuracy of analysts’ profit forecasts. We identified, for example, the importance of optimism, in the second stage of anchoring, but that other biases, such as overconfidence, realism, representativeness, and commonality, although they may impact on accuracy, their importance in this regard is low. And, this can be considered in the prediction models in future research.

We use text analysis to capture some, but not all, cognitive biases from the analysts’ qualitative reports. Text analysis allows those interested in analysts’ reports to be aware of the characteristics of the disclosures, providing a more nuanced view of the reports and the profile of analysts with greater or lesser accuracy. Identifying analysts’ behavioral biases through the narrative of their reports reveals the association between biases and the consequences on analysts’ accuracy, influencing decision-making judgments (Fisher et al., 2019). Accordingly, we used software frequently used in corporate finance, accounting, and psychology literature (Oliveira et al., 2021). However, it is worth noting that the use of text analysis brings with it some elements of subjectivity.

Further, the use of machine learning techniques allowed the study to indicate the negative and positive relationships between behavioral, financial, temporal, and other factors and analyst accuracy but added the possibility of observing the importance or magnitude that these factors have on analyst accuracy. This allowed us to verify that, although some factors were not statistically significant to the analysts’ accuracy, the probabilistic and non-linear relationship between them made it evident that these same factors can be important in determining accuracy, as occurred with volatility, ADR, surprise, loss, popularity, and sector. Thus, a different way of analyzing the data was provided, which allowed for the accuracy of causal and probabilistic dependencies that would not be allowed with the use of linear OLS.

In technical terms, the study highlights the importance of considering non-parametric regression models, which can improve the explanatory power of predictive models. In addition, the research draws attention to the importance of validating models, when applying a study that considers training models followed by testing, making the findings safer as to their validity. Therefore, the techniques applied now differ from what has been used in research, and this is an advance in the field of research.

Considering also a practical contribution of the research, the results obtained open an important discussion for investors, in that the reports of analysts with an optimistic bias are related to a low accuracy; and that reports with anchoring and commonality are related to greater accuracy. Thus, these factors can be taken into account in their valuation models and even in choosing which analysts are to be followed by investors. Still on a practical level, the research indicates brokers the relationship between the analysts’ cognitive biases and their forecast accuracy. As the compensation, image and credibility of brokers depend on the success achieved in profit forecasts, this research highlights the variables and their importance in helping brokers to choose the analysts capable of achieving better accuracy in their profit forecasts.

## Data Availability Statement

The raw data supporting the conclusions of this article may be made available by the authors, upon prior consultation, for use with a scientific nature and for development of research in partnership.

## Author Contributions

PN contributed to the construction and organization of the database, theoretical survey, and statistical analysis and wrote the draft of the manuscript. ER contributed to the definition of the methodology and statistical analysis. JB contributed to theoretical discussions and study design. IA contributed to theoretical discussions and final review of the work. PN, ER, and IA contributed to manuscript revision, read, and approved the submitted version.

## Conflict of Interest

The authors declare that the research was conducted in the absence of any commercial or financial relationships that could be construed as a potential conflict of interest.

## Publisher’s Note

All claims expressed in this article are solely those of the authors and do not necessarily represent those of their affiliated organizations, or those of the publisher, the editors and the reviewers. Any product that may be evaluated in this article, or claim that may be made by its manufacturer, is not guaranteed or endorsed by the publisher.
