# Supplementary material for: The Influence of Cognitive Biases and Financial Factors on Forecast Accuracy of Analysts
Source: Front Psychol. 2022 Jan 4;12:773894. doi: 10.3389/fpsyg.2021.773894 (PMC8764190; doi:10.3389/fpsyg.2021.773894)
Supplement: Supplementary file 1 [file Table_1.doc]

**Appendix**

|  | **Authors** | **Research Objective** | **Country** | **Time** | **Method** | **Results** |
| --- | --- | --- | --- | --- | --- | --- |
|  | **Financial Aspects** | | | | | |
| 1 | Lang & Lundholm (1996) | Analysis between disclosure practices and analysts' forecasts. | USA | 1985-1989 | Linear Regression | Companies with informal policies of secrecy show better analysts' forecasts. |
| 2 | Lang et al. (2002) | Analysis of ADR and Non-ADR Companies. | USA | 1996 | Linear Regression | Companies that issue ADR have more analyst coverage and greater forecasting accuracy. |
| 3 | García-Meca & Sánchez-Ballesta (2006) | Identifying Factors Affecting Analyst Forecast Meta-Analysis. |  |  | Meta-Analysis | Country, Forecast Time Period, Size Influence Analyst Forecast. |
| 4 | Byard et al. (2006) | Examine the association between corporate governance and the quality of information available to the analyst. | USA | 1999-2001 | Linear Regression | Corporate governance is related to the quality of information to be worked on by the analyst. |
| 5 | Kwag & Small (2007) | Analysis of the Impact of Regulation on Analyst Forecast Error. | USA | 1999-2002 | Linear Regression | Analysts are less accurate after fair disclosure regulation. |
| 6 | Tong (2007) | Examining the Impact of Transparency Initiatives on Information Accuracy. | 60 Countries | 1990-2004 | Linear Regression | Disclosure Standards Increase Forecast Accuracy. |
| 7 | Behn, Choi & Kang (2008) | Analyze whether audit quality impacts analyst forecast. | USA | 1996-2001 | Linear Regression | Quality audit impacts analyst forecast. |
| 8 | Ernstberger et al. (2008) | Analyze the influence of international accounting standards on analyst accuracy. | Germany | 1998-2004 | Linear Regression | Accuracy is greater in the use of IFRS than USGAAP. |
| 9 | Xie, Zhang & Zhou (2012) | Examine the relationship between specialization of large auditors and accuracy of analysts. | USA | 1996-2006 | Linear Regression | Companies audited by large auditors show greater accuracy in forecasting analysts. |
| 10 | Abernathy, Hermann & Krishnan (2013) | Examine the association between audit committee expertise and analyst forecast. | USA | 2000-2008 | Linear Regression | Results indicated a positive association between variables. |
| 11 | Dalmácio, Lopes, Rezende & Sarlo Neto (2013) | Analyze the relationship between corporate governance and analyst forecast. | Brazil | 2000-2008 | Linear Regression | Corporate governance positively influences analyst accuracy. |
| 12 | Ayres, Huang, & Myring (2017) | Examine the relationship between the use of fair value accounting and analyst behavior. | USA | 2007-2013 | Linear Regression | Fair value accounting impacts analyst forecast. |
|  | **Economic Aspects** | | | | | |
| 13 | Brown (2001) | Model Definition for More Accurate Analyst Choice. | USA | 1986-1998 | Linear Regression | Focusing on past forecast is important, as are analyst characteristics. |
| 14 | Jacob et al. (1999) | Explore the Effect of Analyst Aptitude and Brokerage Internal Environment on Analyst Accuracy. | USA | 1981-1992 | Linear Regression | Analyst aptitude and brokerage characteristics are associated with forecast accuracy. |
| 15 | Clement (1999) | Analysis of portfolio complexity, resources and capacity interfere with the analyst's accuracy. | Many Countires | 1983-1994 | Linear Regression | Portfolio complexity negatively affects accuracy, while experience affects it positively. |
| 16 | Martinez (2007a) | Investigate the determinants of the accuracy of analysts' forecasts. | Brazil | 1997-2002 | Linear Regression | Analyst experience implies accuracy. |
| 17 | Myring & Wrege (2009) | Understanding factors that explain the variation in analyst accuracy. | USA | 1984-2006 | Linear Regression | The reduction in the analyst's portfolio of companies is related to the increase in its accuracy. |
| 18 | Leuz (2003) | Analysis of the relationship between issuance of ADRs and analyst forecast. | Canadá | 1996 | Linear Regression | Companies that issue ADRs have superior intermediation by analysts. |
| 19 | Bhat, Hope & Kang (2006) | Investigate how difference in transparency affects analyst prediction. | 21 Countries | 1992-2002 | Linear Regression | Transparency is positively related to analyst accuracy. |
|  | **Cognitive Aspects** | | | | | |
| 20 | Amir & Ganzach (1998) | Presentation of a model for analyzing heuristics in analysts' forecasts, observing leniency, representativeness and anchoring. | USA | 1976-1990 | Linear Regression | Analyst tends to overreact on positive forecasts and underreact on negative forecasts. |
| 21 | Easterwood & Nutt (1999) | Analysis of Optimism About Analyst Forecast Inefficiency. | USA | 1982-1995 | Linear Regression | Analysts underreact to negative information, but overreact to positive information. |
| 22 | Lim (2001) | Test a utility function for earnings forecast modeling, where analysts offset bias to improve management access and forecast accuracy. | USA | 1984-1996 | Modeling | Optimal forecasts with minimum expected error are optimistically skewed and exhibit predictable cross-sectional variation related to analyst and firm characteristics.. |
| 23 | Ciccone (2003) | Analyzing Optimism and Analyst Forecasting. | USA | 1977-1993 | Linear Regression | Optimism does not interact with the analyst's forecasting properties by affecting stock returns. |
| 24 | Dittrich et al. (2005) | Analysis of the effects of overconfidence in the investment environment. | Germany |  | Experimento | Overconfidence increases with the absolute deviation from the optimal choices and with the complexity of the task involving the number of risky assets, but decreases with the perceived individual uncertainty. |
| 25 | Baik (2006) | Analysis of the relationship between self-selection bias and analyst forecast. | USA | 1983-2003 | Linear Regression | Companies experiencing financial difficulties, low long-term growth, and falling stock prices appear to be more subject to self-selection by analysts. |
| 26 | Martinez (2007b) | Investigate Optimism in Analysts' Forecast. | | 1995-2002 | Linear Regression | Financial analysts are optimistic in their forecasts. |
| 27 | Marsden, et al. (2008) | Analysis of the influence of heuristics such as representativeness, anchoring and leniency in analyst forecast. | Australia | 1993-2004 | Linear Regression | Analysts make optimistic forecasts, supporting the leniency hypothesis and tend to overreact when forecast revisions and changes are positive and subreact when they are negative. |
| 28 | Campbell & Sharpe (2009) | Check whether the analyst's prediction is influenced by the anchoring bias. | USA | 1992-2006 | Linear Regression | The prediction is influenced by the anchoring bias. |
| 29 | Broihanne et al. (2014) | Analysis of overconfidence in the perception of risk in stock price pricing. | USA | 2011 | Interview | Overconfidence is present in stock price pricing. |
| 30 | Kafayat (2014) | Examine whether investors are prone to irrational behavior when exposed to certain psychological dilemmas related to the financial world and what are the interrelationships between these dilemmas. | Pakistan |  | Questionnaire and Structural Equations | Self-attribution, overconfident and overoptimistic investors make suboptimal decisions and the outcome is less than they expect. |
| 31 | Corredor et al. (2014) | Find out whether the origin of optimism in analysts' forecasts is primarily strategic or whether it also contains an element of cognitive bias. | Europe | 1993-2002 | Linear Regression | Regulation can reduce analyst optimism bias, but benefits are limited by the fact that optimism bias is partially associated with cognitive bias. |
| 32 | Lima & Almeida (2015) | Analyze the performance of the analyst's forecast under the bias of optimism. | Brazil | 2005-2013 | Linear Regression | When analysts make a mistake due to an optimistic forecast, they become less optimistic in their next forecasts. |
| 33 | Galanti & Vaubourg (2017) | Analyze the effect of rules on analysts' forecasts. | France | 1999-2011 | Linear Regression | The analysts' bullish bias has significantly diminished following the French government's rules. |
| 34 | Silva Filho et al. (2018) | Assess the influence of behavioral biases in the formulation of analysts' forecasts. | Brazil | 2000-2015 | Linear Regression | It was concluded that the influence of the leniency and anchoring heuristics was observed, given the optimism verified in the forecast revisions and changes and that forecast errors are greater when the forecast period is far from the earnings reporting period. |
| 35 | Machado (2018) | Observe the influence of heuristics and biases on the detail and tone of analyst reports and how the market reacts to this. | Brazil e USA | 2004-2016 | Linear Regression and Canonical Correlation | Observed that heuristics are present in the reports of financial analysts and that investors react to them. |
|  | **Temporal Aspect** | | | | | |
| 36 | Bandyopadhyay, Brown & Richardson (1995) | Analyze the effect of the forecast horizon on forecasting stock returns by analysts. | Canada | 1983 a 1988 | Linear Regression and Canonical Correlation | When the forecast horizon is close to the reporting period, forecast earnings explain 30% of price change. |
| 37 | Lys & Soo (1995) | Analyze Analyst Forecast Accuracy. | USA | 1980-1982 | Linear Regression | Analyst Accuracy Decreases with Extended Forecast Horizon. |
| 38 | Das & Saudagaram (1998) | Examine the differences in analysts' earnings forecast characteristics. | Many countries | 1984-1989 | Linear Regression | Among the results, it was observed that the accuracy improves with the shorter the forecast horizon. |
| 39 | Jaggi & Jain (1998) | Evaluate Analyst Forecast Accuracy. | Hong Kong | 1993-1994 | Linear Regression | Analyst forecasts are overly optimistic and accurate the shorter the forecast horizon. |
| 40 | Jacob et al. (1999) | Analysis of the relationship between analyst experience and forecast accuracy. | Many countries | 1981-1992 | Linear Regression | Among other things, they noted that a longer forecast horizon leads to less accuracy. |
| 41 | Amiram et al (2017) | Examine whether the analyst's timing, accuracy, and informativeness characteristics of their forecasts change during periods of heightened uncertainty. |  | 1994-2016 | Linear Regression | When uncertainty is high, analysts' forecasts are more timely but less accurate. And yet, in an environment of uncertainty, the punctuality of analysts' forecasts decreases. |
| 42 | Muslu, Mutlu, Radhakrishnan & Tsang (2019) | Analysis of the tone, legibility, length and horizon of the narratives the precision of the analysts. | Many countries | 2000-2011 | Linear Regression | In general, shorter narrative horizons imply greater accuracy. |

Figure 1: Summary of the main research in the project's line of studies
